# Inhibition of lysophosphatidic acid receptor 1 relieves PMN recruitment in CNS via LPA1/TSP1/CXCR2 pathway and alleviates disruption on blood-brain barrier following intracerebral haemorrhage in mice

**DOI:** 10.1186/s12987-023-00434-3

**Published:** 2023-05-10

**Authors:** Ling Gao, Li Peng, Prativa Sherchan, Hong Tang, Yu Liu, Jie Xiao, Hui Shi, Yujie Luo, Jiping Tang, John H. Zhang, Ying Xia

**Affiliations:** 1 Department of Neurosurgery, Affiliated Haikou Hospital, Xiangya School of Medicine, Central South University, Haikou, 570208 China; 2 Department of Physiology and Pharmacology, School of Medicine, Loma Linda University, Loma Linda, CA 92354 USA; 3 Department of Neurosurgery and Anesthesiology, Loma Linda University Medical Center, Loma Linda, CA 92354 USA; 4 Department of Ophthalmology, Central South University Xiangya School of Medicine Affiliated Haikou Hospital, Haikou, Hainan, 570208 China; 5 Department of Ophthalmology, The Second Xiangya Hospital, Central South University, Changsha, 410000 Hunan China

**Keywords:** Lysophosphatidic acid receptor 1, Intracerebral haemorrhage, AM966, Blood-brain barrier, Polymorphonuclear leukocyte

## Abstract

**Backgroud:**

The frequencies of morbidity and impairment associated with spontaneous intracerebral haemorrhage (ICH) are comparatively high. Blood-brain barrier (BBB) integrity was compromised due to subsequent brain injury induced by ICH, which is crucial for a poor prognosis. Polymorphonuclear leukocyte (PMN) strongly modulate the disruption of BBB in the central nervous system (CNS). The lysophosphatidic acid receptor 1 (LPA1) mediated thrombospondin-1 (TSP1) regulation in astrocytes, which induce macrophage inflammatory protein 2(MIP2) secretion. MIP2 enhance PMN recruitment through CXC chemokine type 2 (CXCR2) activation. The purpose of this study was to investigate whether the LPA1-mediated inhibition of PMN recruitment and BBB protection after ICH is regulated by TSP1 and CXCR2 networks.

**Methods:**

ICH induction was performed in CD1 mice using collagenase administration. AM966, a targeted LPA1 antagonist, was orally administered 1 and 12 h following ICH. further identify possible LPA1-mediated BBB protection mechanisms, we intracerebroventricularly (ICV) administered a CXCR2 ligand MIP2, as well as TSP1 CRISPR activation (ACT) with AM966. Consequently, we performed neurobehavioral, brain water content (BWC), Evans blue staining (EBS), immunofluorescence (IF), and western blot (WB) analyses.

**Results:**

After ICH, astrocytes showed signs of LPA1, which peaked after 24 h, while PMN\ displayed evidence of CXCR2. The AM966-mediated LPA1 suppression relieved PMN recruitment, diminished brain oedema, demonstrated extravasation (as evidenced by EBS), protected BBB integrity, and enhanced neurologic activity following ICH. AM966 treatment strongly reduced TSP1, CXCR2, Occludin, and Claudin-5 expressions and PMN recruitment following ICH, and their expressions were restored by MIP2 and TSP1 CRISPR (ACT).

**Conclusions:**

This study shows that LAP1 suppression reduced PMN recruitment after ICH in mice via TSP1/CXCR2 signalling, which minimized BBB disruption and improved the CNS’s neurobehavioral functioning. Hence, LPA1 is a strong candidate for therapy to reduce PMN recruitment and offer protection of BBB integrity after ICH.

**Supplementary Information:**

The online version contains supplementary material available at 10.1186/s12987-023-00434-3.

## Background

Spontaneous intracerebral hemorrhage (ICH) is a type of stroke caused by blood extravasating into the cerebral parenchyma. It is associated with relatively high rates of morbidity and disability [1]. Approximately 35 ~ 52% of patients expire within 30 days of ICH, resulting in a relatively poor long-term prognosis [2, 3]. The recent pandemic study has shown that ICH incidence has increased and is now showing up in a growing percentage of younger people. However, there are few effective treatments for recovering from this challenging illness [4]. ICH-induced secondary brain injury, induced by neuroinflammation, impaired blood-brain barrier (BBB), brain oedema, and cellular apoptosis, is an important contributor to poor patient prognosis [5]. BBB is a unique system that modulates the transfer of substances between brain tissue and blood, and its primary function is to protect the central nervous system (CNS) [6]. BBB disruption induces neutrophil invasion, which, in turn, aggravates inflammation. In the meantime, neuroinflammation impairs BBB integrity, thereby causing brain oedema [7]. According to one study, brain oedema expands and is most evident on the first day following ICH [8]. More studies are focusing on BBB protection to improve brain function following ICH [9–11]. Prior work suggested that leukocytes are present in the cerebrospinal fluid and peri-hematoma area 6 h after ICH, and leukocyte infiltration enhances brain oedema [12]. Multiple researches revealed that the polymorphonuclear leukocyte (PMN) recruitment has a strongly connection with BBB disruption in several neurological diseases [13–16]. PMN are the primary modulators of BBB disruption, and PMN deficiency restores BBB integrity, and minimizes astrocyte loss during neuroinflammation [17]. A potential treatment strategy for protecting the BBB following ICH is PMN recruitment.

Lysophosphatidic acid(LPA)is a bioactive phospholipid, which, when enriched in the brain, promotes multiple biological functions via interaction with its six receptors [18]. The Lysophosphatidic acid receptor 1(LPA1) involved in numerous biological metabolic processes, such as, cell proliferation, survival, apoptosis, and proinflammation within the CNS [19]. Our previous investigation revealed that LPA1 suppression attenuates neuroinflammation following ICH, Interestingly, we also demonstrated that LPA1 suppression effectively diminishes brain oedema and neutrophil infiltration [12]. However, it is still unclear if LPA1 suppression preserves BBB integrity following ICH. Hisaoka-Nakashima and his team reported that LPA1 activation stimulates thrombospondin-1 (TSP1) secretion via the ERK, MAPK, JNK signaling network in astrocytes [20]. TSP1 is an extracellular matrix protein that physically associates with a myriad of ligands, namely, cell receptors, growth factors, cytokines, and proteases to modulate a numerous physio- and pathological activities [21, 22]. Previous study revealed that TSP1 induces blood-brain barrier leakage in mice, which upregulates the release of macrophage inflammatory protein 2 (MIP-2) and other chemokines [23, 24]. Moreover, the CXC chemokine type 2 (CXCR2) expression enhances PMN recruitment by MIP2 releasing. TSP1 was found to be elevated following ICH [25], but it is still unclear how this affected PMN recruitment. Furthermore, it is unknown whether LPA1 impacts BBB integrity through controlled TSP1 expression after ICH. The present study hypothesized that LPA1 inhibition would protect BBB integrity by reducing PMN recruitment via the LPA1/TSP1/CXCR2 signalling pathway following ICH in mice. We investigated the putative LPA1 antagonist AM966-mediated attenuation of BBB disruption and associated restoration of neurological function in ICH mice to understand further the role of the LPA1 receptor in the ICH-mediated BBB disruption.

## Materials and methods

### Mice

In all, 141 8-week-old male CD1 mice, weighing between 30 and 40 g were obtained from Charles River, USA, and housed separately for ≥ 3 days prior to ICH surgery, with open access to food and water. The mice were arbitrarily separated into experimental groups. The experimental design, groups, and mice per group are presented in Supplementary Figure S1 and Supplementary Table S1. All animal protocols received ethical approval from Loma Linda University and followed the National Institutes of Health guidelines. In addition, we followed the ARRIVE (Animal Research: Reporting of In Vivo Experiments) reporting criteria.

### ICH establishment

As reported previously, the ICH model was established [26]. Briefly, mice were treated with ketamine/xylazine (100/10 mg/kg) intraperitoneally, and upon attaining deep anaesthesia, they were placed face down in a stereotactic head frame (Kopf Instruments, Tujunga, CA). Drilled a 1 mm cranial burr incision 1.5 mm right lateral to the midline and 0.9 mm posterior to the bregma and inserted a 27-gauge needle into the right basal ganglia for 4 mm below the dura mater. Collagenase (0.075 U diluted with saline 0.5 µL volume, VII-S; Sigma Aldrich, St. Louis, MO) was administered using a micro infusion pump (Harvard Instruments, Holliston, MA) at a rate of 0.25 µL/min. Sham mice underwent identical operation, however, they were given 0.5 µL saline, instead of ICH-causing collagenase. Following administration completion, the needle was maintained for an additional 10 min to minimize administered solution extravasation. Subsequently, bone wax sealed the burr hole, following which, the needle was removed, and the skin was sutured to facilitate the mouse’s recovery. Using a heating blanket, the body temperature of mice was kept constant at 37 ± 0.5 °C.

### Experimental protocols

#### Experiment 1

Following ICH, we analyzed the LPA1, TSP1, and CXCR2 expressions in the right hemisphere of mice via WB at time points 6, 12, 24, and 72 h, as well as 7 days (n = 6). To determine the LPA1 sub-cellular localization within astrocytes, we employed immunofluorescence (IF) staining for 24 h following ICH (n = 2). Along with LPA1, we also stained for glial fibrillary acidic protein (GFAP); with CXCR2, we also stained for Neutrophil Elastase (NE). Control mice underwent sham surgery (SS).

#### Experiment 2

Ly6G/CD11b IF staining was employed to assess the Lpa1 selective antagonist AM966 (Advanced ChemBlocks, USA, 30 mg/kg) [12] induced LPA1-mediated suppression of PMN recruitment 24 h after ICH. Mice were randomly placed in one of 3 groups (n = 6), namely, SS, ICH + Vehicle (DMSO, ICH + V), and ICH + AM966 (ICH + A).

#### Experiment 3

We then evaluated BBB integrity after AM966 suppressed LPA1. Mice (n = 6) were divided into one of three groups at random: SS, ICH + V(DMSO), and ICH + A. Among them, AM966 and vehicle were provided via oral gavage at 1 and 12 h post-ICH. The brain water content (BWC) was evaluated at 24 h post ICH. Additionally, the Evans Blue staining (EBS) and IF of CD31/ZO1 were examined 24 h post ICH.

#### Experiment 4

To further examine the mechanism involving the LPA1/TSP1/CXCR2 signaling pathway, we employed MIP2 and TSP1 CRISPR activation(ACT). We injected 2 µl [27] of corresponding CRISPRs (Santa Cruz Biotechnology, USA) into mouse via ICV administration 48 h prior ICH. MIP2 (Sigma-Aldrich, USA) 0.01 µg [28, 29]was given via ICV administration 1 h after ICH. Mice were randomly grouped as follows (n = 6): SS, ICH + V, ICH + A, ICH + A + PBS, ICH + A + MIP2, ICH + A + CRISPR control, and ICH + A + TSP1 CRISPR. WB analysis was conducted 24 h post ICH.

### Assessment of motor activity

Modified Garcia, forelimb placement (FP), and corner turn examinations were blinded, as reported in an earlier publication [30]. In short, the Modified Garcia test was a combination of 7 distinct assessments of spontaneous, whisker touch, side stroke, limb symmetry, forelimb walking, lateral turning, and climbing activities. We used the FP test to quantify the left forepaw placements out of 20 stimulations. Animals were positioned at the vertex of a 30° arena, and the left turn quantity was documented out of 10 trials.

### BWC assessment

BWC was measured as described in a prior publication [31]. In short, following mice sacrifice, brain samples were extracted immediately, prior to dissection into the left/right basal ganglia, left/right cortices, and cerebellum. All dissected portions of the brain were weighed using analytical balance (Denver Instrument, USA) to achieve the respective wet weights (WW). The dry weight (DW) was measured after samples were baked for 24 h in an oven at 100 °C. BWC was computed as follows: BWC (%) = [(WW − DW)/WW] × 100%.

### EBS extravasation

EBS extravasation was conducted as reported previously [32]. To prepare 4% EBS dye, EBS powder (Sigma-Aldrich, USA) was re-suspended in normal saline. Then, it was intraperitoneally administered (4 mL/kg) to mice, followed by a 3 h incubation. Subsequently, mice were placed under deep anesthesia, the heart was perfused with 50 mL of ice-cold phosphate buffered saline (PBS), and the brains were excised and split into left and right hemispheres, prior to storage in − 80 °C freezer until additional analyses. Each brain specimen was added to 1100 µL PBS, followed by homogenization, sonication, and centrifugation (4 °C, 14,000 rcf, 30 min) to obtain the supernatant. Before the second centrifugation (4 °C, 14,000 rcf, 30 min), trichloroacetic acid (TCA) was added to produce the supernatant. Using a spectrophotometer (Thermo Fisher, USA), the EBS concentration was measured at 610 nm and then quantified using the standard curve.

### Western blot

The western Blot (WB) protocol was described previously [33]. In short, mice were anesthetized, prior to perfusion with ice-cold PBS. Brains were excised, followed by the separation of the two hemispheres. Before being centrifuged at 14,000 g for 30 min at 4 °C, the right hemispheres were homogenized in RIPA lysis buffer (Santa Cruz Biotechnology, USA). Protein isolates from the resulting supernatant were combined with loading buffer, electrophoresed on SDS-PAGE, transferred to nitrocellulose membrane, blocked for two hours in blocking buffer, and then exposed for an overnight (ON) period at 4 °C to primary antibodies listed in Supplementary Table S2. Matched secondary antibodies were introduced for 1.5 h at room temperature (RT), then protein visualization was done with enhanced chemiluminescent (ECL) Plus kit (Amersham Biosciences, USA) and an imaging system (Bio-Rad, Versa Doc, model 4000). Protein quantification employed Image J (NIH, USA).

### IF assessment

As previously stated in publications, the IF analysis was conducted [34]. Anesthetized mice underwent perfusion with ice-cold PBS, then brains were excised, prior to fixation in 10% formalin, followed by a 24 h dehydration in 30% sucrose formalin, and slicing to 10 μm sections via CM3050S cryostat (Leica Biosystems, Germany). The prepared sections were next ON exposed to primary antibodies summarized in Supplementary Table S2 at 4 °C. Next, the sections were washed in PBS before a 2 h exposure to secondary antibodies at RT, with subsequent PBS-rinse and DAPI staining. Staining visualization employed a fluorescence microscope (Leica Microsystems, Germany).

### Statistical analysis

Graph Pad Prism (San Diego, USA) was used for all statistical data analysis. The data are expressed as mean ± SD. Multiple comparisons were performed with one-way ANOVA and two-way repeated measures ANOVA analysis by Tukey post hoc test, p < 0.05 was set as the significance threshold.

## Results

### Mice ortality and elimination

The total mortality rate was 4.4% (5/113) among ICH mice, and 0% (0/28) among sham mice. When all experimental mice were compared, there were no noticeable variations in mortality rates. None of the model was eliminated.

### Temporal LPA1, TSP1, and CXCR2 expressions

**Fig. 1:**
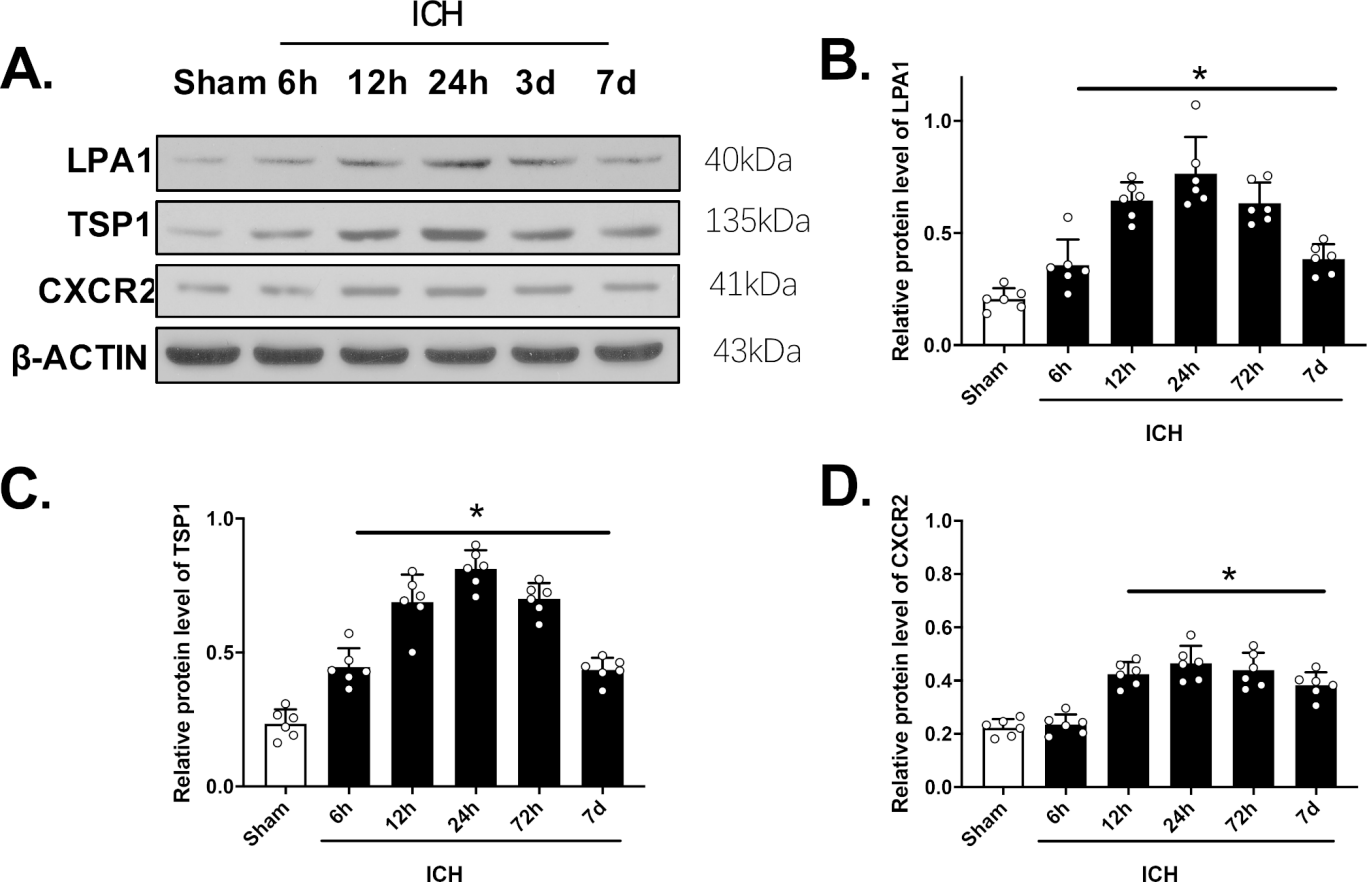
Western blot (WB) analysis of LPA1; TSP1 and CXCR2 expression of time course in the ipsilateral hemispheres following intracerebral hemorrhage (ICH). Representative band images **(A)** and quantitative analysis of LPA1 **(B)**; TSP1 **(C)**; CXCR2 **(D)**; One-way ANOVA, Tukey’s test, mean ± SD (error bars). * p < 0.05 vs. sham. n = 6 per group

**Fig. 2:**
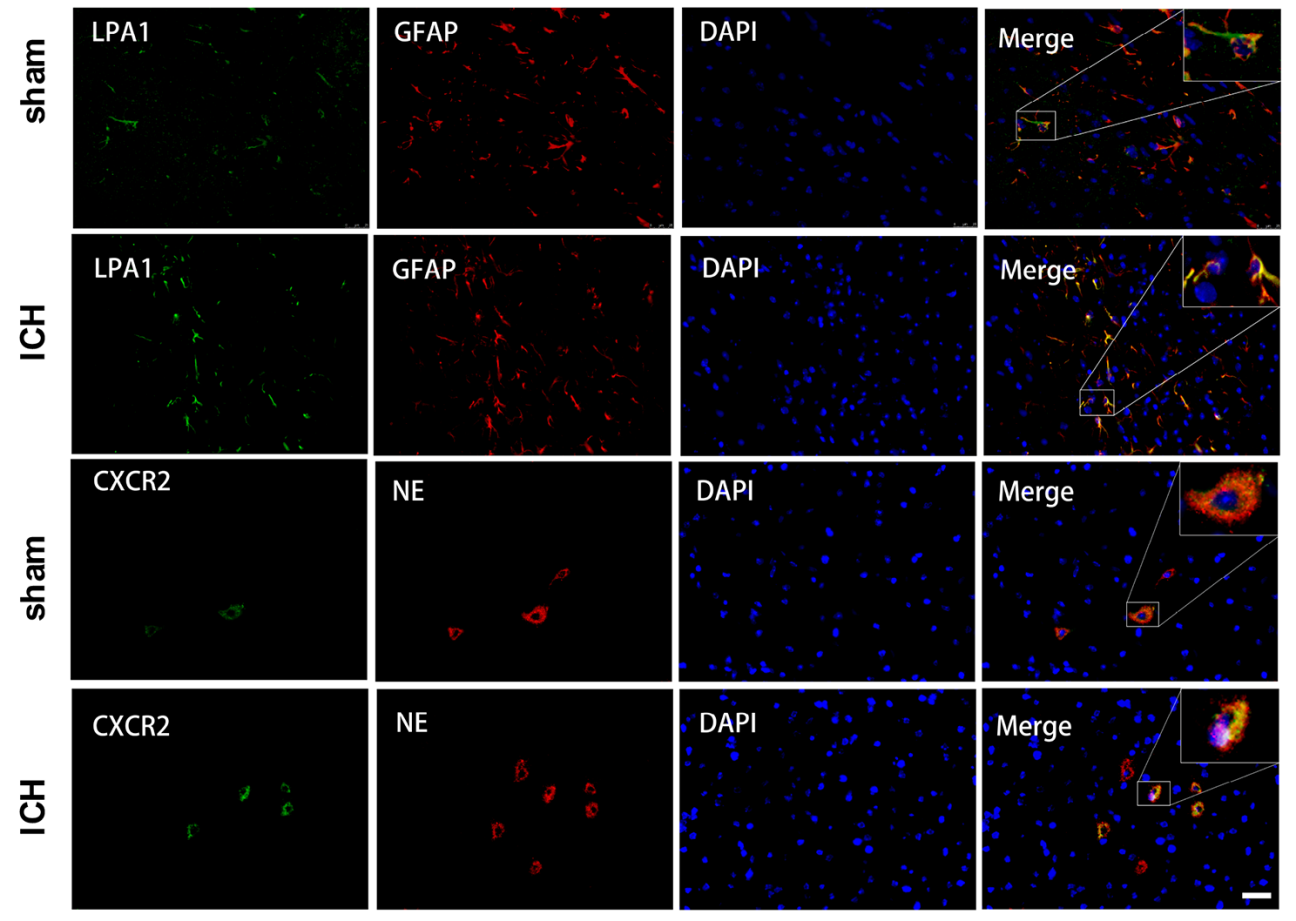
Expression of LPA1 and CXCR2 in cells at 24 h following ICH. Representative Immunofluorescence images of expression of LPA1 in astrocyte (GFAP), and expression of CXCR2 in PMN (NE) in the perihematomal region. (Scale bar = 25 μm)

The right hemisphere LPA1, TSP1, and CXCR2 expressions at 6, 12, 24, and 72 h, as well as 7 days following ICH were assessed via WB analysis. Based on our results, all three protein expressions were enhanced, and peaked at 24 h following ICH, relative to the sham mice (p < 0.05, Fig. 1A–D). Using double IF staining, we demonstrated significant co-localization of LPA1 with glial fibrillary acidic protein (GFAP) in astrocytes and CXCR2 with neutrophil elastase (NE) in PMN 24 h after ICH and in sham mice (Fig. 2).

### AM966 treatment attenuated PMN recruitment

**Fig. 3:**
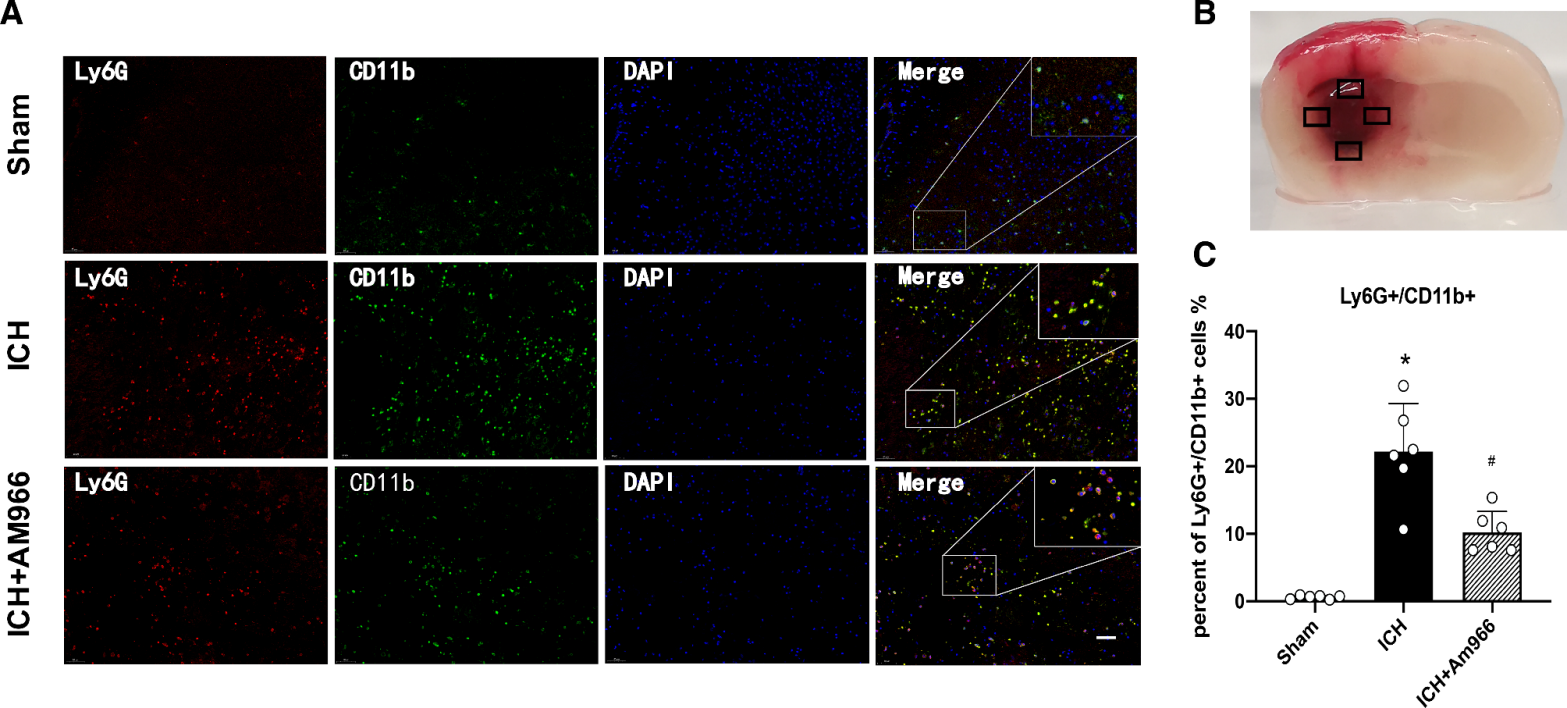
AM966 reduced the recruitment of PMN perihematomal region at 24 h post-ICH. **(A)** Representative images of immunofluorescence staining of Ly6G (red) and CD11b (green) in the perihematomal region at 24 h post-ICH (Scale bar = 50 μm). **(B)** Brain sample with identification of the areas (marked with box) used for Ly6G/ CD11b positive cell counting in the perihematomal region. C, Quantitative analyses of Ly6G/ CD11b -positive cells in the perihematomal region at 24 h post- ICH D. One-way ANOVA, Tukey’s test, mean ± SD (error bars). n = 6 per group *P < 0.05 vs. sham group; #P < 0.05 vs. ICH + Vehicle group

PMN recruitment was examined within the perihematomal region using LY6G/CD11b-based IF staining (Fig. 3A). Based on our results, AM966 exposure strongly diminished PMN recruitment, compared to vehicle exposure 24 h following ICH (p < 0.05, Fig. 3B).

### LPA1 inhibition by AM966 reduced BBB disruption after ICH

**Fig. 4:**
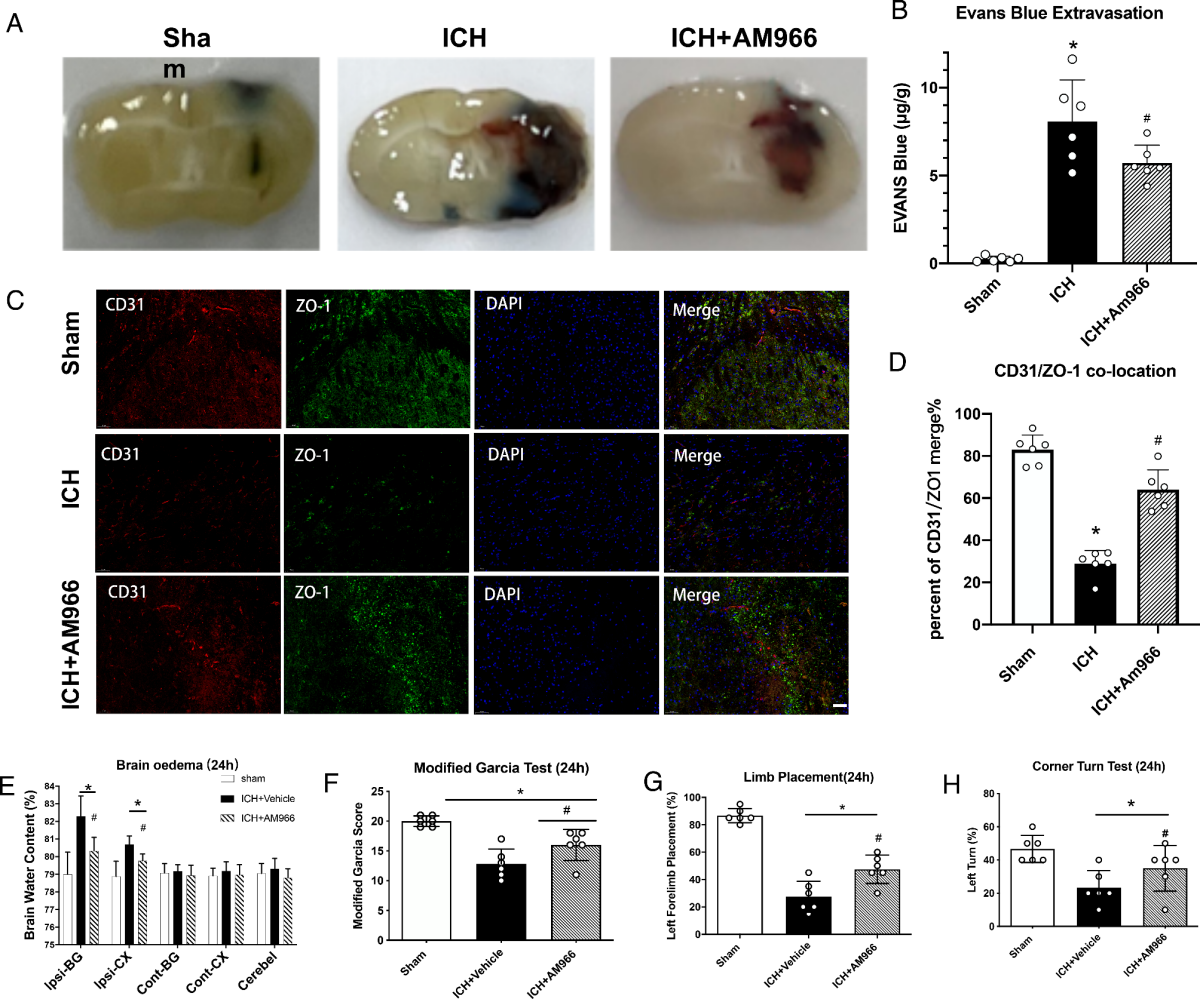
Representative images of Evans Blue staining **(A)**; Evans Blue Extravasation analyses **(B)**; Representative images of immunofluorescence staining of CD31 (red) and ZO-1 (green) in the perihematomal region **(C)** and Quantitative analyses of CD31/ ZO-1 positive cells in the perihematomal region **(D)** at 24 h post-ICH. The effects of AM966 on brain water content **(E)**; modified Garcia score **(F)**; Limb Placement score **(G)** and Corner Turn score **(H)**; One-way ANOVA, Tukey’s test, mean ± SD (error bars), (B; D; F-H) and two-way repeated measures ANOVA, Tukey’s test, mean ± SD (error bars) **(E)**, n = 6 per group. *p < 0.05 vs. sham group; #p < 0.05 vs. ICH + Vehicle group. Ipsi-BG ipsilateral basal ganglia, Ipsi-CX ipsilateral cortex, Cont-BG contralateral basal ganglia, Cont-CX contralateral cortex, Cerebel cerebellum

BBB integrity was assessed via EBS and IF staining of CD31/ZO-1. Relative to the vehicle-treated mice, EBS extravasation around hematoma was markedly enhanced at 24 h following ICH (p < 0.05), and were strongly diminished upon AM966 administration (p < 0.05, Fig. 4A, B). The CD31 and ZO-1 overlaps around the hematoma were strongly diminished at 24 h following ICH (p < 0.05), and were elevated upon AM966 administration (p < 0.05, Fig. 4C, D).

### AM966 induced LPA1 inhibition mitigated brain oedema and enhanced neurological function following ICH

BWC was examined at 24 and 72 h following ICH. In comparison to vehicle mice, the right basal ganglia and cortex BWC were markedly enhanced at 24 h, and 72 h (p < 0.05), respectively, and were strongly diminished upon AM966 administration (p < 0.05, Fig. 4E). AM966 restored neurobehavioral function at 24 and 72 h following ICH (p < 0.05, Fig. 4F-H).

### MIP 2 reversed the AM966-mediated protection of BBB integrity

**Fig. 5:**
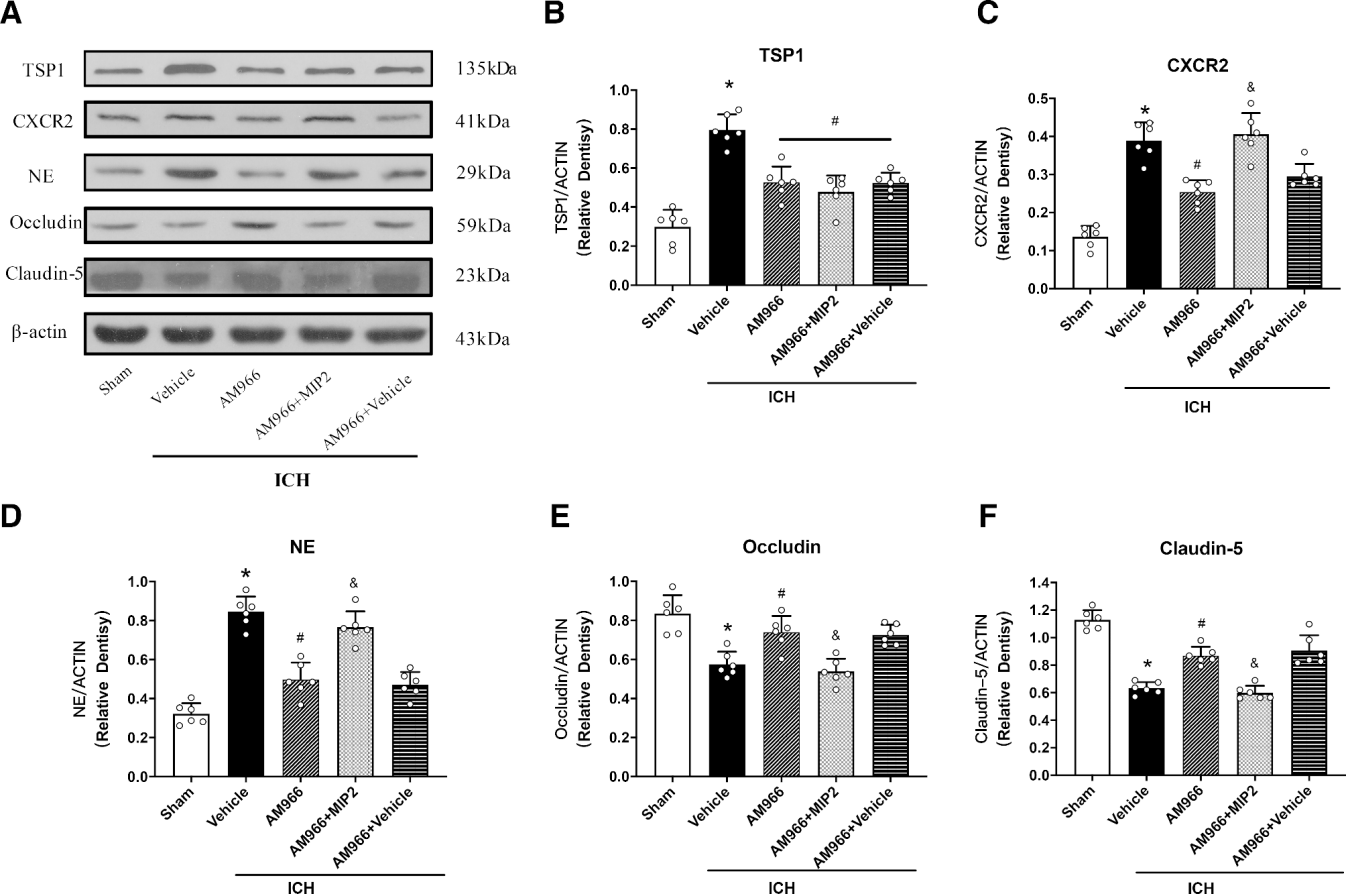
The effects of AM966 and CXCR2 ligand MIP2 on the expression of TSP1 and downstream signaling proteins. a Representative western blot bands. B–F Quantitative analyses of TSP1, CXCR2, NE, Occludin, Claudin-5 in the ipsilateral hemisphere at 24 h after ICH. *p < 0.05 vs. sham, #p < 0.05 vs. ICH + V, and &p < 0.05 vs. ICH + AM966 + V One-way ANOVA, Tukey’s test, mean ± SD (error bars) n = 6 per group

Figure 5 depicts the strong decrease in TSP1, CXC2R, Occludin, and Claudin-5 expressions, as well as PMN recruitment following AM966 administration (p < 0.05), and this process was reversed upon CXC2R ligand MIP2 administration, which upregulated CXCR2 expression, and enhanced PMN recruitment while decreasing Occludin and Claudin-5 expressions at 24 h following ICH (p < 0.05, Fig. 5A-F).

### TSP1 activation reversed the AM966-mediated protection of BBB integrity

**Fig. 6:**
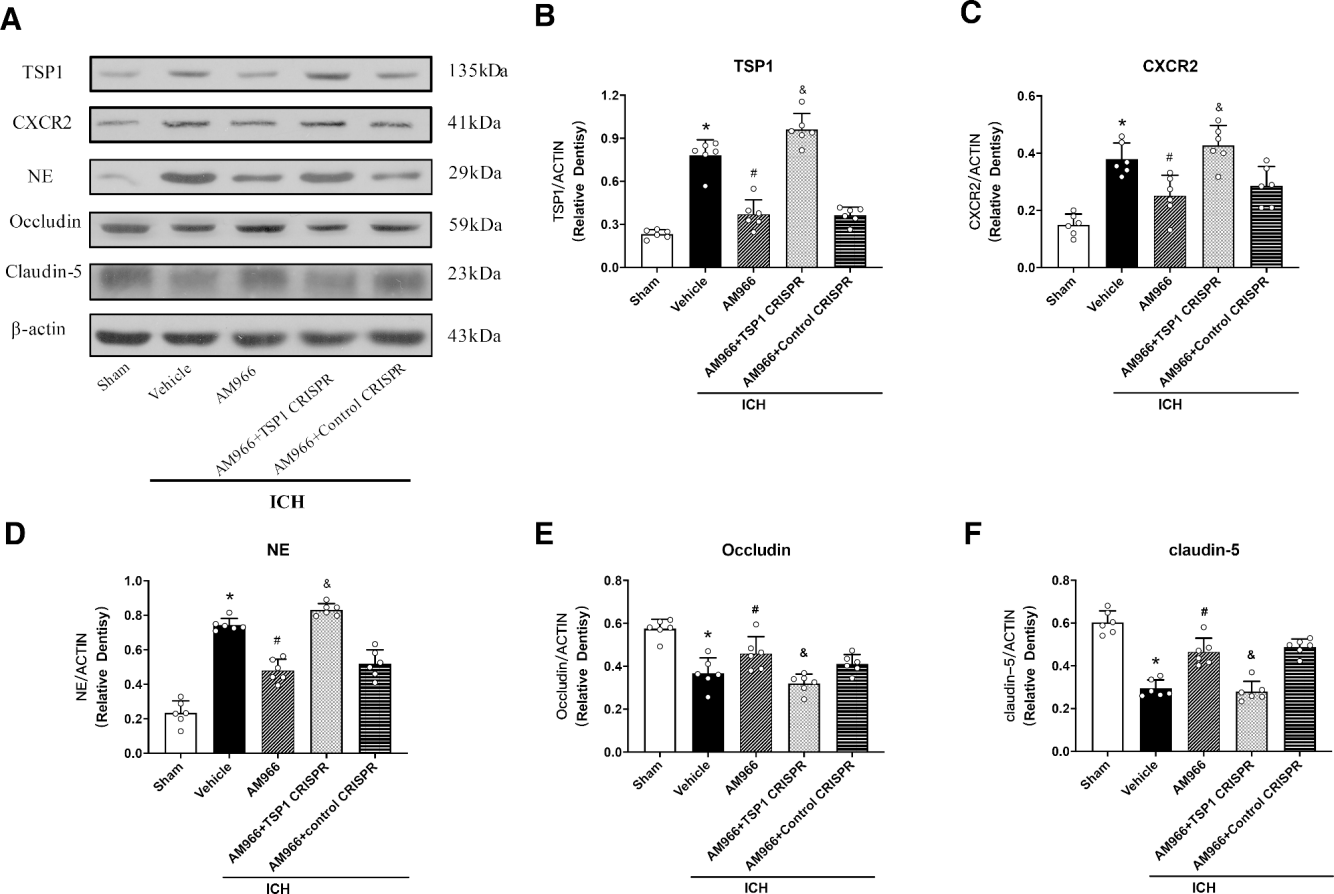
The effects of AM966 and TSP1 CRISPR (ACT) on the expression of TSP1 and downstream signaling proteins. a Representative western blot bands. B–F Quantitative analyses of TSP1, CXCR2, NE, Occludin, Claudin-5 in the ipsilateral hemisphere at 24 h after ICH. *p < 0.05 vs. sham, #p < 0.05 vs. ICH + V, and &p < 0.05 vs. ICH + AM966 + control CRISPR. One-way ANOVA, Tukey’s test, mean ± SD (error bars) n = 6 per group

We employed TSP1 CRISPR activation plasmid (TSP1 CRISPR ACT) to stimulate TSP1 expression to examine its underlying mechanisms. Figure 6 illustrates that the TSP1, CXC2R, Occludin, and Claudin-5 expressions, as well as PMN recruitment were strongly diminished following AM966 treatment (p < 0.05). Moreover, TSP1 CRISPR (ACT) upregulated TSP1 expression, consistently enhanced CXCR2 expression, and augmented PMN recruitment, while decreasing Occludin and Claudin-5 expressions (p < 0.05, Fig. 6A-F).

## Discussion

An earlier study revealed that inhibiting LPA1 reduces neuroinflammation and brain edema in rodents suffering from ICH. This study investigated whether LPA1 inhibition mitigated BBB disruption following ICH in mice and elucidated the mechanism underlying this phenomenon. Based on our analysis, AM966-induced LPA1 inhibition strongly diminished brain oedema and PMN recruitment to enhance BBB integrity in the perihematomal region following ICH. Moreover, we investigated the mechanism behind the AM966-mediated BBB protection, and revealed that AM966 exposure strongly reduced TSP1 release, and deactivated the downstream CXCR2, NE, Occludin and Claudin-5 expressions. In contrast, the CXCR2 ligand reversed the AM966-induced effects. Similarly, TSP1 activation via TSP1 CRISPR (ACT) also produced the same impact. These data indicated that LPA1 inhibition attenuates BBB disruption by downregulating PMN recruitment via the TSP1 /CXCR2 network following ICH.

LPA receptors are ubiquitous in the CNS. Among them, LPA1 contributes heavily to the LPA network. Emerging reports suggested that LPA induces TSP1 synthesis in cortical astrocytes via LPA1 receptor activation [20].TSP1 and MIP2 are upregulated in PMN recruitment [35], MIP2 recruits PMN, and activates the CXCR2 receptor. Likewise, we also revealed that the LPA1 content in the haemorrhage hemisphere reached its peak at 24 h following ICH. More`over, both TSP1 and CXCR2 increased upon LPA1 upregulation. In addition, our double IF staining revealed that LPA1 was co-localized with astrocytes, confirming our earlier findings. In the meantime, our results indicated that CXCR2 co-localized with PMN, a phenomenon markedly amplified after ICH. There are additional accounts of ICH causing BBB disruption through secondary brain injury [36], and PMN are critical in this process [37]. LPA1 inhibition was used to reduce neuroinflammation and neutrophil invasion in our prior study, and this approach results in significant improvements in brain oedema and mice outcome following ICH using a selective antagonist for LPA1, AM966. Herein, we demonstrated that AM966 strongly downregulated PMN recruitment and diminished BWC following ICH. Similarly, using CD31/ZO-1 IF staining and EBS extravasation analysis, we revealed that the BBB disruption was diminished upon AM966 administration. We speculated that AM966 administration potentially diminished PMN recruitment, ultimately improving BBB integrity.

We also examined the underlying mechanism behind the LPA1-mediated enhancement of BBB integrity following ICH. Based on emerging evidence, LPA enhances TSP1 release by activating LPA1 in astrocytes [20]. TSP1 is a regulator of specific chemokines, namely, JE and MIP-2, which can increase PMN recruitment via upregulation of the CXCR2 receptor expression [23, 24, 38]. Herein, we demonstrated that AM966 exposure markedly reduced TSP1 and CXCR2 expressions, as well as PMN recruitment while upregulating Occludin and Claudin-5 expressions following ICH. MIP2, a CXCR2 ligand, administration markedly enhanced brain tissue CXCR2 content as well as PMN recruitment while reversing the potential benefits of AM966. To confirm the importance of this network in BBB protection, we used TSP1 CRISPR (ACT) to activate TSP1 before AM966 administration, and the AM966-mediated positive effects were reversed with TSP1 CRISPR (ACT) administration. This evidence revealed that inhibiting LPA1 via the LPA1/TSP1/CXCR2 network protects BBB integrity.

This research has certain limitations. First, we only examined PMN recruitment-mediated BBB disruption following LAP1 suppression. The BBB disruption pathophysiology following ICH is rather complex, and LPA1 is known to activate Rho kinase [39, 40], which is also involved in BBB disruption [41]. Therefore, we cannot exclude that the BBB protection seen in our experiments was not caused by activated Rho kinase. Second, prior investigations by other scientists as well as our team revealed ubiquitous LPA1 content in the CNS [42, 43], and LPA1 may be a mediator of cellular death, which requires exploration in future research. Third, we used 6 mice per group in our experiment, maybe affected the power of the result, but it is still statistically significant. To enhance the reliability, we will increase the number of animals in further research. Finally, herein, we employed EBS extravasation to evaluate BBB integrity. EBS extravasation generally demonstrates only large molecular weight substances (MWSs) [44]. Hence, we did not assess the possible transfer of small MWSs across the BBB following ICH.

## Conclusion

Based on our analyses, AM966-mediated LPA1 inhibition markedly reduced PMN recruitment via the LPA1/TSP1/CXCR2 network, thereby enhancing BBB integrity and attenuating brain oedema in an in vivo ICH model. Given these evidences, LPA1 targeting may be a robust treatment approach for minimizing PMN recruitment and protecting BBB in ICH patients.

## Electronic supplementary material

Below is the link to the electronic supplementary material.

Supplementary Material 1

Supplementary Material 2

Supplementary Material 3

Supplementary Material 4

## Data Availability

The datasets used and/or analyzed in the current study are available from the corresponding authors on request.

## Abbreviations

ICH: Intracerebral hemorrhage
BBB: Blood brain barrier
CNS: Central nervous system
PMN: Polymorphonuclear leukocyte
LPA: Lysophosphatidic acid
LPA1: LPA receptor 1
TSP1: Thrombospondin-1
CXCR2: CXC chemokine type 2
