# Supplementary material for: Inhibition of lysophosphatidic acid receptor 1 relieves PMN recruitment in CNS via LPA1/TSP1/CXCR2 pathway and alleviates disruption on blood-brain barrier following intracerebral haemorrhage in mice
Source: Fluids Barriers CNS. 2023 May 10;20:33. doi: 10.1186/s12987-023-00434-3 (PMC10173532; doi:10.1186/s12987-023-00434-3)
Supplement: Supplementary file 1 — Supplementary Material 1 [file 12987_2023_434_MOESM1_ESM.pdf]

Experimental Groups	BWC	WB	IF	EB	death	Mortality	Shared
**Experiment 1**							
Sham	0	6	2	0		0%(0/8)	0
ICH (6h,12h,24h,72h,7d)	0	30	2	0	1	2.9%(1/33)	0
**Experiment 2**							
Sham	0	0	6	0		0%(0/6)	6
ICH + Vehicle	0	0	6	0		0%(0/6)	6
ICH + AM966	0	0	6	0	1	14.3%(1/7)	6
**Experiment 3**							
Sham	6	0	0	6		0%(0/12)	IF Share with Exp.2
ICH + Vehicle	6	0	0	6	1	7.7%(1/13)	IF Share with Exp.2
ICH+AM966	6	0	0	6		0%(0/12)	IF Share with Exp.2
**Experiment 4**							
Sham	0	6	0	0		0%(0/6)	0
ICH + Vehicle	0	6	0	0		0%(0/6)	0
ICH + AM966	0	6	0	0		0%(0/6)	0
ICH + AM966 + MIP2	0	6	0	0	1	14.3%(1/7)	0
ICH + AM966+ Vehicle	0	6	0	0		0%(0/6)	0
ICH + AM966+Control CRISPR	0	6	0	0		0%(0/6)	0
ICH + AM966+TSP1 CRISPR (ACT)	0	6	0	0	1	14.3%(1/7)	0
						ICH 4.4%(5/113)	
TOTAL	18	78	22	18	5	3.5%(5/141)	

Supplementary Tab S1. Animal use in each experimental group.
