# Supplementary material for: Inhibition of lysophosphatidic acid receptor 1 relieves PMN recruitment in CNS via LPA1/TSP1/CXCR2 pathway and alleviates disruption on blood-brain barrier following intracerebral haemorrhage in mice
Source: Fluids Barriers CNS. 2023 May 10;20:33. doi: 10.1186/s12987-023-00434-3 (PMC10173532; doi:10.1186/s12987-023-00434-3)
Supplement: Supplementary file 2 — Supplementary Material 2 [file 12987_2023_434_MOESM2_ESM.pdf]

Western blot antibody
primary antibodies:<br>rabbit anti-LPA1 (1:1000, LifeSpan Biosciences, Seattle, WA);<br>rabbit anti-TSP1 (1:1000, Abcam, Cambridge, MA);<br>rabbit anti-CXCR2 (1:1000, Thermofisher , Waltham, MA);<br>rat anti-NE (1:1000, Abcam, Cambridge, MA);<br>rabbit anti-Occludin (1:1000, Abcam, Cambridge, MA);<br>rabbit anti-Claudin-5 (1:1000, Abcam, Cambridge, MA);<br>mouse anti-actin (1:5000, Santa Cruz Biotechnology, Dallas, TX).
secondary antibodies<br>Goat Anti-Rabbit IgG (1:3000, Santa Cruz Biotechnology, Dallas, TX);<br>Goat Anti-Mouse IgG (1:3000, Santa Cruz Biotechnology, Dallas, TX);<br>Goat Anti-Rat IgG (1:3000, Santa Cruz Biotechnology, Dallas, TX).
Immunofluorescence staining antibody
primary antibodies:<br>rabbit anti-LPA1 (1:100, LifeSpan Biosciences, Seattle, WA);<br>goat anti-GFAP (1:100, Abcam, Cambridge, MA);<br>rabbit anti-CXCR2 (1:50, Thermofisher , Waltham, MA);<br>rat anti-NE (1:50, Abcam, Cambridge, MA);<br>rat anti-Ly6G(1:50, Santa Cruz Biotechnology, Dallas, TX).<br>rabbit anti-CD11b (1:50, Abcam, Cambridge, MA).<br>rabbit anti-CD31 (1:100, Abcam, Cambridge, MA).<br>rat anti-ZO-1 (1:100, Santa Cruz Biotechnology, Dallas, TX).
secondary antibodies<br>Donkey Anti-Rabbit IgG(H+L) (1:200, Jackson Immunoresearch, West Grove, PA)<br>Donkey Anti-Rat IgG(H+L) (1:200, Jackson Immunoresearch, West Grove, PA)<br>Donkey Anti-Goat IgG(H+L) (1:200, Jackson Immunoresearch, West Grove, PA)

Supplementary Tab S2. Antibody use in Western blot and Immunofluorescence staining.
