# Supplementary material for: Inhibition of lysophosphatidic acid receptor 1 relieves PMN recruitment in CNS via LPA1/TSP1/CXCR2 pathway and alleviates disruption on blood-brain barrier following intracerebral haemorrhage in mice
Source: Fluids Barriers CNS. 2023 May 10;20:33. doi: 10.1186/s12987-023-00434-3 (PMC10173532; doi:10.1186/s12987-023-00434-3)
Supplement: Supplementary file 3 — Supplementary Material 3 [file 12987_2023_434_MOESM3_ESM.pdf]

Experiment Design and groups

Experiment 1:Time course of LPA1,TSP1and CXCR22, cellular co-localization of LPA1 and CXCR2

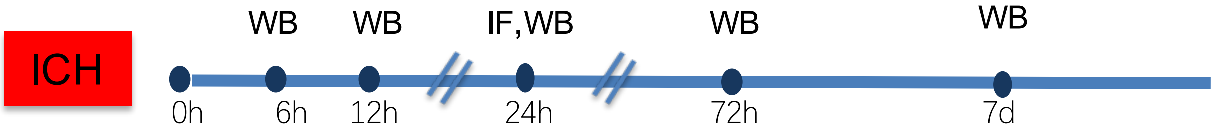

Exp1. Groups:
1)Sham
2)ICH 6h
3)ICH 12h
4)ICH 24h
5)ICH 72h
6)ICH 7d

Experiment 2: PMNs recruitment affected by AM966

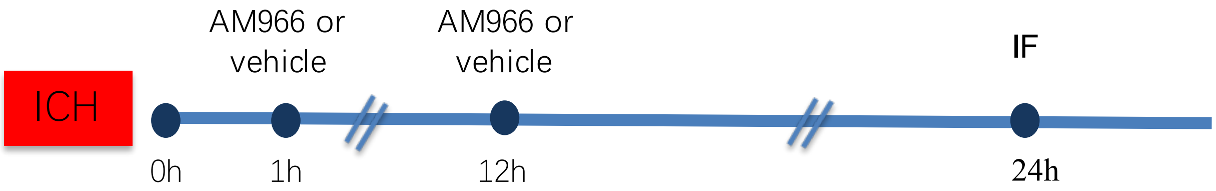

Exp2. Groups:
1) Sham
2) ICH + Vehicle
3) ICH + AM966

Experiment 3 : Neurobehavioral Functions , Brain Edema affected and BBB Protection effect by AM966

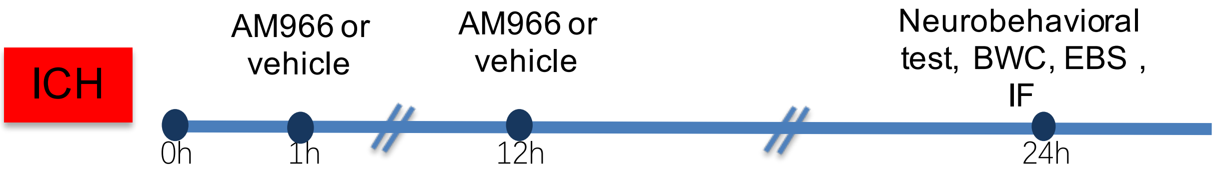

Exp3. Groups :
1) Sham
2) ICH + Vehicle
3) ICH + AM966

Experiment 4: Pathway of BBB protection effect by AM966 after ICH

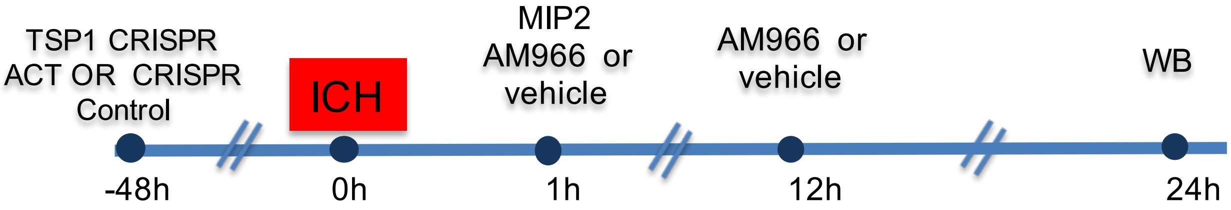

Exp4. Groups
1) Sham
2) ICH + Vehicle
3) ICH + AM966
4) ICH + AM966+ Vehicle
5) ICH + AM966+ MIP2
6) ICH + AM966+ TSP1 CRISPR(ACT)
7) ICH + AM966+ CRISPR Control
