# Supplementary material for: Inhibition of lysophosphatidic acid receptor 1 relieves PMN recruitment in CNS via LPA1/TSP1/CXCR2 pathway and alleviates disruption on blood-brain barrier following intracerebral haemorrhage in mice
Source: Fluids Barriers CNS. 2023 May 10;20:33. doi: 10.1186/s12987-023-00434-3 (PMC10173532; doi:10.1186/s12987-023-00434-3)
Supplement: Supplementary file 4 — Supplementary Material 4 [file 12987_2023_434_MOESM4_ESM.docx]

**Figure1**

LPA1
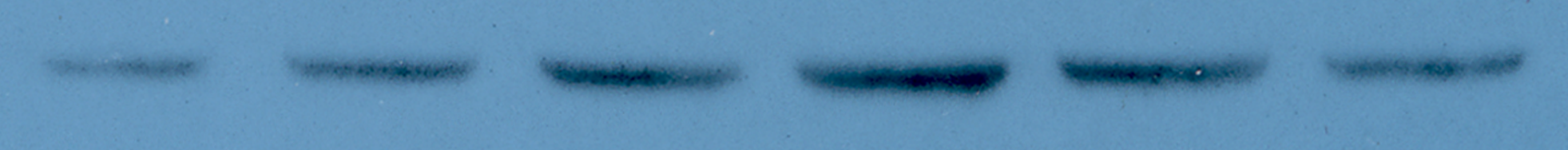
 40 kDa

TSP1
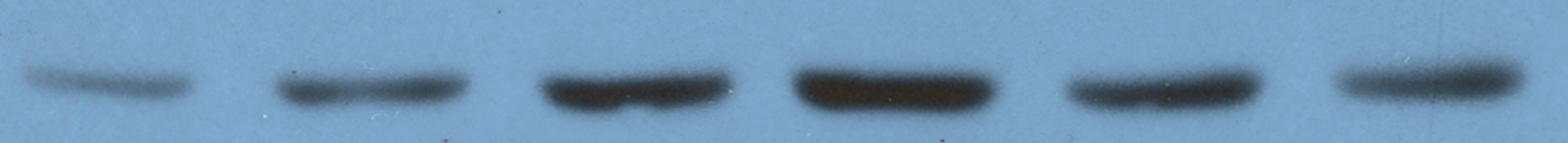
 135 kDa

CXCR2
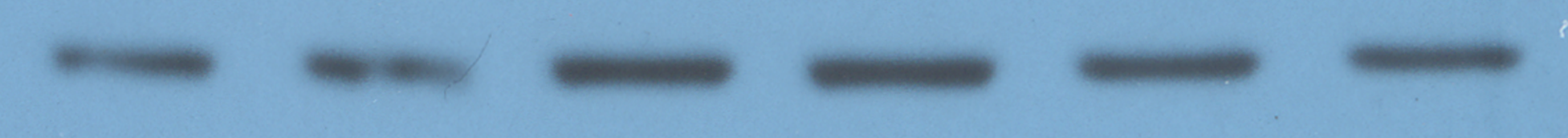
 41 kDa

Actin
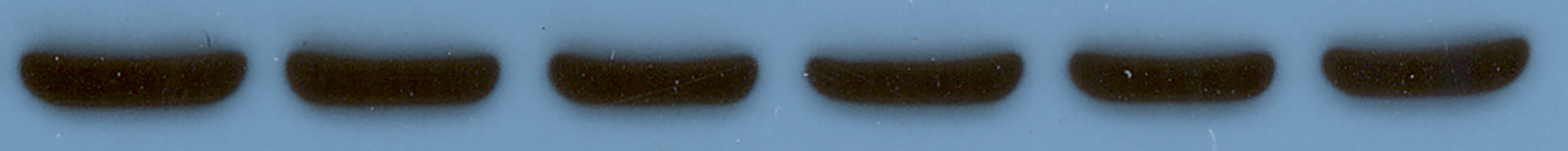
 43 kDa

**Figure5**

TSP1
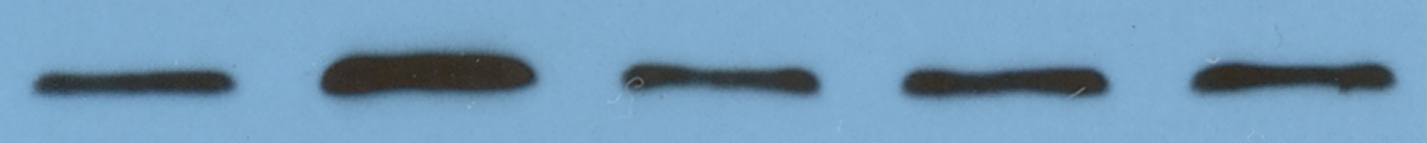
135 kDa

CXCR2
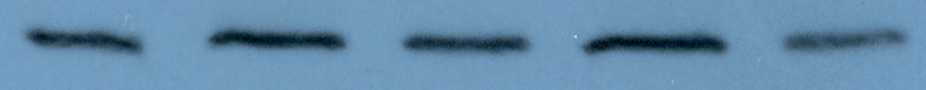
41 kDa

NE
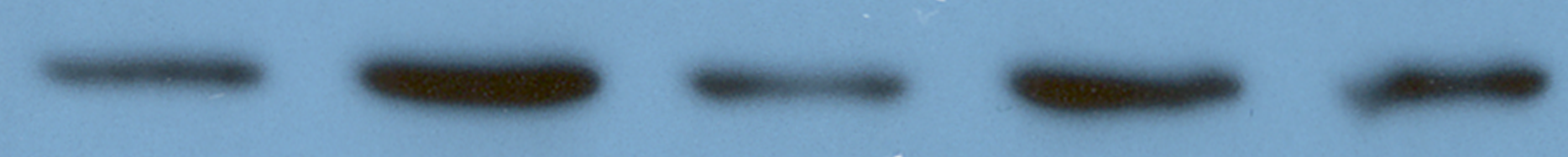
29 kDa

Occludin
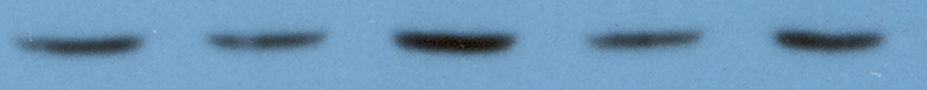
59 kDa

Claudin-5
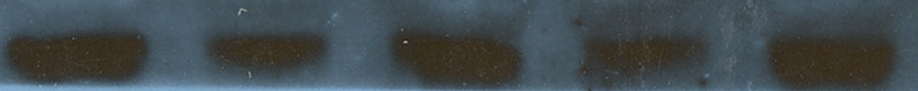
23 kDa

Actin
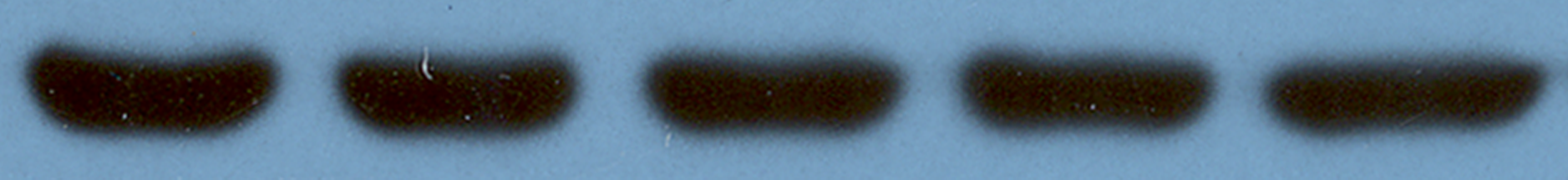
43 kDa

**Figure6**

TSP1
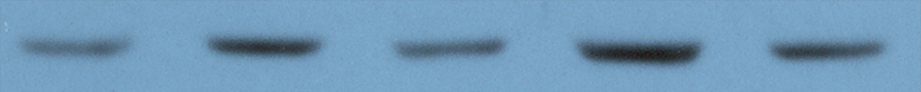
135 kDa

CXCR2
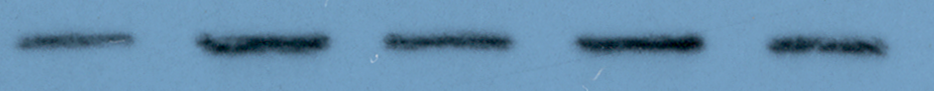
41 kDa

NE
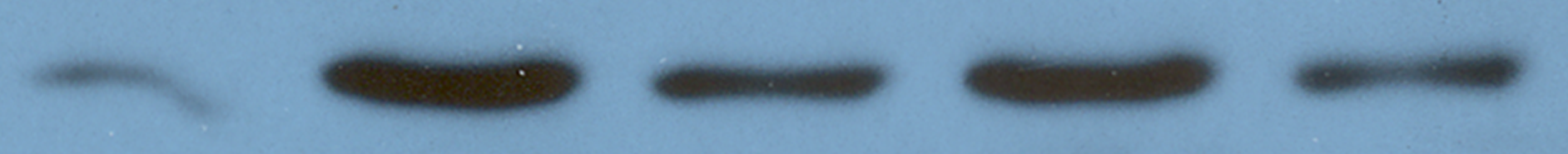
29 kDa

Occludin
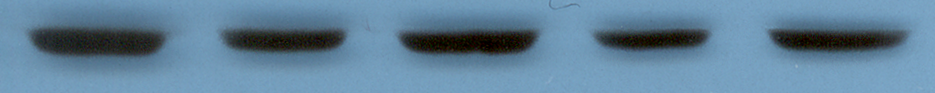
59 kDa

Claudin-5
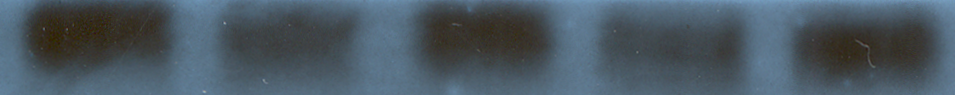
23 kDa

Actin
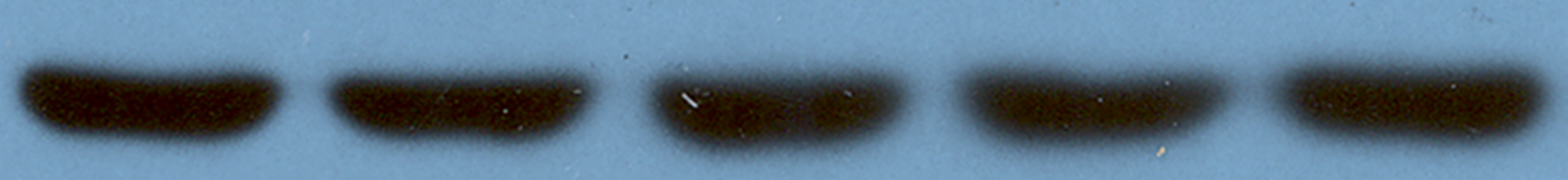
43 kDa

LPA1 full flim TSP1 full film

CXCR2 full film NE full film

Occludin full film Claudin-5 full film

Actin full film
